# Physiological and Biogeochemical Traits of Bleaching and Recovery in the Mounding Species of Coral *Porites lobata*: Implications for Resilience in Mounding Corals

**DOI:** 10.1371/journal.pone.0063267

**Published:** 2013-05-02

**Authors:** Stephen J. Levas, Andréa G. Grottoli, Adam Hughes, Christopher L. Osburn, Yohei Matsui

**Affiliations:** 1 The School of Earth Sciences, The Ohio State University, Columbus, Ohio, United States of America; 2 Scottish Association for Marine Science, Oban, Scotland, United Kingdom; 3 Chemistry Division, Naval Research Laboratory, Washington DC, United States of America; University of New South Wales, Australia

## Abstract

Mounding corals survive bleaching events in greater numbers than branching corals. However, no study to date has determined the underlying physiological and biogeochemical trait(s) that are responsible for mounding coral holobiont resilience to bleaching. Furthermore, the potential of dissolved organic carbon (DOC) as a source of fixed carbon to bleached corals has never been determined. Here, *Porites lobata* corals were experimentally bleached for 23 days and then allowed to recover for 0, 1, 5, and 11 months. At each recovery interval a suite of analyses were performed to assess their recovery (photosynthesis, respiration, chlorophyll *a*, energy reserves, tissue biomass, calcification, δ^13^C of the skeletal, δ^13^C, and δ^15^N of the animal host and endosymbiont fractions). Furthermore, at 0 months of recovery, the assimilation of photosynthetically acquired and zooplankton-feeding acquired carbon into the animal host, endosymbiont, skeleton, and coral-mediated DOC were measured via ^13^C-pulse-chase labeling. During the first month of recovery, energy reserves and tissue biomass in bleached corals were maintained despite reductions in chlorophyll *a*, photosynthesis, and the assimilation of photosynthetically fixed carbon. At the same time, *P. lobata* corals catabolized carbon acquired from zooplankton and seemed to take up DOC as a source of fixed carbon. All variables that were negatively affected by bleaching recovered within 5 to 11 months. Thus, bleaching resilience in the mounding coral *P. lobata* is driven by its ability to actively catabolize zooplankton-acquired carbon and seemingly utilize DOC as a significant fixed carbon source, facilitating the maintenance of energy reserves and tissue biomass. With the frequency and intensity of bleaching events expected to increase over the next century, coral diversity on future reefs may favor not only mounding morphologies but species like *P. lobata*, which have the ability to utilize heterotrophic sources of fixed carbon that minimize the impact of bleaching and promote fast recovery.

## Introduction

Coral reefs are declining globally due to a combination of direct and indirect human impacts. Increases in seawater temperature are of primary concern as they lead to mass coral bleaching events [1], a phenomenon where whole communities of corals loose a significant portion of their vital endosymbiotic algae (*Symbiodinium* spp.) and/or their algal photosynthetic pigments [1]–[5]. Mass bleaching events are expected to increase in frequency and intensity in the coming decades [4], [6], [7], and are already causing mass coral reef decline worldwide [8]. Coral bleaching susceptibility has been directly linked to the magnitude of thermal stress [9], irradiance levels [10], symbiont types [11], thermal history of the site [12]–[14], and coral morphology [15]–[18]. Despite evidence for higher survival rates among mounding and encrusting forms of corals post bleaching relative to branching species [16], [17], [19]–[21], the physiological and biogeochemical trait(s) that may underlie mounding and encrusting coral resilience are not well understood. Yet, determining what trait(s) confers resilience in mounding species of coral is central to understanding how coral reef structure and diversity might change in the future [18].

Coral acquire carbon (C) two ways: photoautotrophically and heterotrophically. Healthy corals are able to meet 100% of their daily metabolic requirements photoautotrophically [22]–[25] and between 5–40% heterotrophically [24]–[28]. Photoautotrophic C is used primarily to meet metabolic demand and for calcification, whereas heterotrophic (zooplankton acquired) C is used primarily for tissue building and growth [29]. When bleached, photosynthesis decreases [24], [30]–[32] resulting in a dramatic decrease in the amount of photosynthetically fixed C translocated to the host from the endosymbiont [29]. Thus, bleached corals must rely on one or more of the following mechanisms to survive during recovery: a) catabolism of energy reserves (i.e., lipids, carbohydrates, proteins) [24], [30], [32]–[35], b) increased zooplankton feeding [24], [25], [27], and c) reduced metabolic demand. For example in Hawaii, the branching coral *Montipora capitata* is able to meet at least 150% of metabolic demand by increasing feeding when bleached [24], [25], whereas *Porites compressa* cannot, and relies completely on energy reserves after bleaching and throughout recovery until its endosymbiont populations recover.

Furthermore, changes in the host tissue, algal endosymbiont, and skeletal stable carbon isotopic signature during bleaching and recovery are diagnostic of the relative contribution of photosynthesis and heterotrophy to the coral [36]. When heterotrophy increases relative to photosynthesis, the animal tissue stable carbon isotopic composition (δ^13^C) becomes depleted [36], whereas decreases in photosynthesis result in depleted endosymbiont and skeletal δ^13^C signatures [36]–[39]. Similarly, the stable nitrogen isotopic signatures (δ^15^N) of bleached and recovering coral animal host and endosymbiont fractions have been shown to track the inorganic and organic sources of nitrogen to the coral holobiont [36], [40], [41].

However, the vast majority of the physiological and biogeochemical information we have on bleached corals, particularly in the Pacific, is derived from branching corals. Yet while mounding corals are often perceived to be resilient to bleaching (i.e., [15]), the traits that drive that resilience are not well characterized (i.e., [15], [18], [42]–[44]). Furthermore, as van Woesik et al. 2011 [18] suggests, establishing what shared and unique traits that confer resilience to bleaching in mounding and branching corals is essential to projecting how coral reef assemblages will change in the future. Thus, to determine how mounding corals are managing to recover from bleaching and persist on reefs, we conducted a comprehensive study of the physiological and biogeochemical traits of the coral animal host, algal endosymbiont, and skeleton in the mounding coral *Porites lobata* immediately following bleaching and throughout a year of recovery. Such a comprehensive study only exists for two branching species of Hawaiian coral: *Montipora capitata* and *Porites compressa* [24], [32], [36]. Furthermore, we assessed how C that is acquired photoautotrophically and heterotrophically (via zooplankton and dissolved organic carbon (DOC)) is assimilated and utilized by the coral holobiont. DOC represents a large pool of C in coral reef seawater (reviewed in Dinsdale and Rohwer (2011) [45]), however DOC as a source of fixed C has never been evaluated in bleached coral and could play a pivotal role in coral persistence. Finally, *P. lobata* is one of the most ubiquitous mounding species of coral in the Pacific Ocean [46] and is a predominant coral reef framework builder in the Eastern Pacific [47]–[49]. As such, it is representative of a large group of important corals whose specific short and long-term physiological and biogeochemical responses to bleaching are not fully understood.

In the present study, the following variables were measured in bleached and non-bleached *P. lobata* corals immediately following bleaching and throughout 11 months of recovery: photosynthesis (P), respiration (R), chlorophyll *a* (Chl *a*), total soluble lipid, soluble animal protein, soluble animal carbohydrate, tissue biomass, calcification, δ^13^C of the three main coral fractions (skeleton, animal host, and endosymbiotic algae), δ^15^N values of the animal host and endosymbiont, the acquisition and allocation of photosynthetically and heterotrophically derived C by all three main coral fractions, the uptake and release of DOC by corals, and the source of C used for DOC release by corals. Collectively, these measurements were used to identify the trait or group of traits that underlie(s) resilience. Our data are one of the first to provide evidence that bleached corals utilize DOC as a significant fixed C source. Furthermore we show that resilience in the mounding coral *P. lobata* is driven by harboring the thermally tolerant C15 endosymbiont, the immediate catabolism of heterotrophically derived C, and the utilization of DOC as a fixed C source.

## Materials and Methods

**Figure 1:**
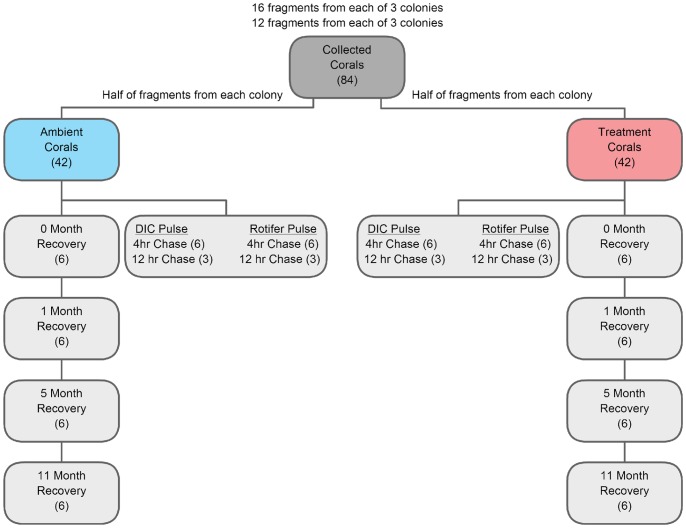
Diagram of coral collection and experimental design. Six genetically distinct coral colonies were fragmented with half of the fragments from each colony placed into ambient tanks (control) and the other half placed into treatment tanks. After 23 days at experimental conditions corals were either immediately collected (0 month recovery corals), pulse-chased with either DIC or Rotifer (only at 0 month recovery), or placed back on the reef to recover and subsequently collected after 1, 5, and 11 months. Numbers in parentheses indicate how many coral fragments that were collected at each step.

*Porites lobata* is a mounding species of coral ranging from yellow-brown to dark brown in color. On 11 August 2006 under special activity permits SAP 2007–28 and SAP 2008–4 issued by the Hawaii Department of Land and Natural Resources, 6 healthy colonies of *P. lobata* were identified at 10–12 m depth in the Sanpan Channel in Kaneohe Bay, Hawaii (21°26.18′N, 157°47.56′W). Sixteen fragments were collected from each of three of the colonies and another 12 coral fragments were collected from each of the other three colonies for a total of 84 coral fragments (Fig. 1). All fragments were mounted onto pre-labeled tiles and placed in outdoor flow-through seawater tanks shaded with two layers of neutral density mesh to mimic light conditions at the recovery site (3 m). After two weeks of acclimation to tank conditions at ambient temperatures, each fragment was buoyantly weighed and then half of the fragments from each colony were randomly placed in one of four treatment tanks (30.2±0.20°C) (42 fragments) and the other half where placed into one of four ambient control tanks (27.5±0.08°C) (42 fragments) from 18 August 2006 to 9 September 2006 for a total of 23 days (Fig. 1). Treatment tank temperatures were gradually raised over the course of a week using industrial heaters to mimic the rate of warming observed on these Hawaiian reefs at the onset of bleaching events. Fragments were rotated within tanks and among tanks of the same temperature to minimize any possible positional effects within and among tanks.

After 23 days, one treatment and one control fragment per colony was then collected and frozen for a total of 12 fragments (6 treatment and 6 control). In addition, 36 fragments (four treatment and four control fragments from each of three colonies and two treatment and two control fragments from each of the remaining three colonies) were used in a pulse-chase labeling experiment as follows: 9 treatment and 9 control fragments (18 total fragments) were pulse-chase labeled with ^13^C-labeled dissolved inorganic C (DI-^13^C) and the remaining 9 treatment and 9 control fragments (18 total fragments) were pulse-chase labeled with ^13^C-labeled rotifers (^13^C-rotifer) (details below) then frozen for δ^13^C analyses (Fig. 1). None of these coral fragments had the opportunity to recover from the experimental conditions and are henceforth referred to as corals with 0 months of recovery. The remaining 36 fragments (18 treatment and 18 control fragments) were placed back onto the reef to recover at three meters depth. One treatment and one control fragment per colony was collected after 1 month (7 October 2006) (6 treatment and 6 control), 5 months (4 Feb 2007), and 11 months (20 August 2007) of recovery (Fig. 1). Throughout the study, all samples were frozen, shipped, and stored at −80°C at The Ohio State University for analysis unless otherwise noted.

At each recovery interval, all collected fragments were buoyantly weighed and gross P, day R, and night R rates were independently measured in three treatment and three control fragments. All collected fragments were analyzed for Chl *a*, total soluble lipid, soluble animal protein, soluble animal carbohydrate, tissue biomass, δ^13^C of the skeleton, and the natural δ^13^C and δ^15^N of the animal and endosymbiont fraction (see analytical methods details below).

### ^13^C-Labeling Experiment

The pulse-chase labeling procedure is described in detail in Hughes et al. (2010) [29]. In summary, to track the acquisition and allocation of C via the photoautotrophic pathway, bleached and non-bleached control fragments were placed into continuously aerated 40 l aquaria filled with 25 l of seawater and pulse-labeled with DI-^13^C for 8 hrs during the day on 20 September 2006. At 8am, 4.5 ml of 0.117M-98 atom% ^13^C NaHCO_3_ was added to one bleached coral-containing aquarium, one non-bleached control coral-containing aquarium, and one seawater control aquarium (containing no corals), yielding an average initial aquarium seawater δ^13^C_DIC_ value of 878.16‰ ±8.72 (1SE) [29]. After 8 hrs, coral fragments were removed from the incubation aquaria and returned to unlabeled, natural flow-through seawater tanks. One fragment from each colony was collected after a chase interval of 4 hrs, and the remaining 2 to 3 fragments were collected after a chase interval of 12 hrs. δ^13^C-DIC in each aquarium at the beginning (888.8‰ and 661.46‰) and end of the 8 hr incubation (708.2‰ and 565.5‰) indicate that the seawater remained highly labeled throughout the incubation.

To track the acquisition and allocation of C via the heterotrophic pathway from zooplankton, additional bleached and non-bleached control fragments were placed into continuously aerated 40 l aquaria filled with 16 l of seawater and were pulse-labeled with ^13^C-rotifer for 10 hrs at night on 20 September 2006. At 8pm, ^13^C-labeled rotifers were added to one bleached coral-containing aquarium, one non-bleached control coral-containing aquarium, and one seawater control aquarium (containing no corals) at a density of 10–15 rotifers per ml of seawater. The rotifers had been ^13^C-labeled by feeding them ^13^C-labeled *Nanocropsis* paste for 96 hrs prior to the pulse incubation. Rotifer δ^13^C values averaged 3985.51‰ ±756.08 (1SE). The corals and controls were incubated with ^13^C-Rotifer for 10 hrs. Before dawn, the corals were removed from the incubation aquaria and returned to unlabeled, natural flow-through seawater tanks. One bleached and one non-bleached fragment from each colony was collected after a chase interval of 4 hrs, and 3 more fragments were collected after a chase interval of 12 hrs.

To track the production and uptake of DOC, 220ml-filtered seawater samples were taken from each tank (bleached, non-bleached, control) 8 hrs after the DI-^13^C pulse incubation and 9 hrs after the ^13^C-rotifer pulse incubation. Seawater samples were collected using a peristaltic pump and filtered at 0.45 µm into pre-cleaned 250ml poly-carbonate bottles and immediately frozen at −50°C, shipped to the US Naval Research Laboratory, then analyzed for DOC concentration and δ^13^C-DOC according to methods below.

### Laboratory Analyses

#### Photosynthesis and Respiration, Chlorophyll a, Energy Reserves, and Calcification

P and R rates were measured on at least three individual bleached and non-bleached control *P. lobata* fragments after 1, 5, and 11 month recovery intervals and standardized to surface area (cm^2^) according to methods in Grottoli et al. (2006) [24]. P and R measurements were not made after 0 months of recovery because these data already existed for Hawaiian *P. lobata* from Grottoli et al. (2006) [24] (standardized to grams ash free dry weight (gdw)) where the experimental location, collection site, coral population, experimental design, and treatments were identical to those used in the current study. Chl *a*, total soluble lipid, soluble animal protein, and soluble animal carbohydrate concentrations were each measured on a 0.75cm diameter coral plug from each fragment. Each measurement was made on whole coral samples (skeleton+animal tissue+endosymbiont) ground with a mortar and pestle and normalized to total ash-free dry tissue biomass of the organic fraction (animal tissue+endosymbiont). Chl *a* was extracted using methods modified from Jeffrey and Humphrey (1975) [50]. Total soluble lipids were extracted using methods described in Rodrigues and Grottoli (2007) [32], while soluble animal carbohydrate and protein concentrations were measured using the methods modified from Dubois et al. (1956) [51], and Smith et al. (1985 ) [52], respectively, as described in Rodrigues and Grottoli (2007) [32]. Calcification was determined using the buoyant weight technique [53]. Surface areas of each fragment were determined using the foil technique [54]. Calcification is reported as mg calcium carbonate produced per day per cm^2^. Therefore, any differences between non-bleached and bleached fragments are independent of colony size. Calcification rates were not measured at 5 and 11 months recovery.

#### Stable Isotopic Analyses

A full description of these methods is given in Hughes et al. (2010) [29]. In summary, coral fragments were airbrushed to remove all tissue from the skeleton. The tissue and endosymbionts were separated by centrifugation and the skeleton sampled by shaving only the very top 0.1 mm or less of each fragments. Animal host and endosymbiont δ^13^C values (δ^13^C_h_ and δ^13^C_e_, respectively) were determined using a Costech Elemental Analyzer where the resulting CO_2_ gases were analyzed for δ^13^C with a Finnigan Delta IV stable isotope ratio mass spectrometer (IRMS). δ^13^C_h_ and δ^13^C_e_ values were reported relative to Vienna Peedee Belemnite Limestone standard (VPDB) (δ^13^C = per mil deviation of the ratio of stable C isotopes ^13^C:^12^C relative to VPDB). In addition, the difference between δ^13^C_h_ and δ^13^C_e_ was calculated to determine the relative contribution of heterotrophically- to photosynthetically-acquired fixed C [37]. Host tissue and endosymbiont fraction δ^15^N values (δ^15^N_h_ and δ^15^N_e_, respectively) were reported relative to air (δ^15^N = per mil deviation of the ratio of stable nitrogen isotopes ^15^N:^14^N relative to air). Repeated measurements of internal standards (n = 24) had a standard deviation of ±0.08‰ for organic δ^13^C and ±0.15‰ for organic δ^15^N. Skeleton δ^13^C values (henceforth δ^13^C_s_) were determined using an automated Kiel III carbonate device coupled to a Finnigan Delta IV IRMS and reported relative to VPDB standard. Repeated measurements of internal standards (n = 51) had a standard deviation of ±0.03‰ for inorganic δ^13^C. δ^13^C pulse-chase experiment values of the animal host, endosymbiont, and the skeletal fractions were reported as per-mil enrichment relative to the average of the natural abundance (i.e., non-labeled) isotopic abundance values from the zero month recovery bleached and non-bleached control fragments.

#### DOC concentration and δ^13^C-DOC

Seawater DOC concentration and δ^13^C-DOC were measured at the US Naval Research Laboratory using methods described in Osburn and St-Jean (2007) [55]. In summary, DOC concentrations were measured by wet chemical oxidation on an OI Analytical 1010 TOC analyzer. The δ^13^C-DOC value of the resulting CO_2_ gas was measured by an in-line Thermo Finnigan Delta^Plus^ XP. Repeated measurements of the concentration and δ^13^C of internal DOC standards had a standard deviation of ±6 µmol and ±0.40‰, respectively. DOC concentrations were reported relative to that of the seawater control aquarium values and standardized to the total surface area of all fragments present in each aquarium. Likewise, the bleached and non-bleached δ^13^C-DOC values were reported as enrichments relative to the baseline values of the seawater control aquarium δ^13^C-DOC.

#### Statistical Analyses

Three-way analysis of variance (ANOVA) was used to test the effect of temperature, recovery interval, and genotype on P, day R, night R, Chl *a*, total soluble lipid, soluble animal protein, soluble animal carbohydrate, calcification rate, δ^13^C_s_, δ^13^C_h_, δ^13^C_e_, δ^13^C_h_–δ^13^C_e_, δ^15^N_h_, and δ^15^N_e_ values. Temperature was fixed with two levels (bleached and non-bleached control), recovery interval was fixed with 4 levels (0, 1, 5, and 11 months recovery), and genotype was random. All data were tested for normal distribution from plots of the residuals versus the predicted values for each variable. Only δ^13^C_e_ data were non-normal and were natural log transformed to achieve normality prior to ANOVA analysis. Bonferroni corrections were not applied due to increased likelyhood of false negatives [56], [57], therefore significant model *p*-values should be interpreted with caution. Furthermore, we realize that multiple ANOVAs without Bonferroni corrections have inherent limitations, but are more informative and have fewer weaknesses than using a Bonferroni correction or using multivariate approaches with this dataset. A posteriori slice tests were used to determine if bleached and non-bleached control averages significantly differed from each other within each recovery interval. The bleached coral fragments were determined to be fully recovered for a given variable once the average bleached value no longer significantly differed from the non-bleached control. Having all six colony genotypes in each treatment at each recovery interval minimized any genotypic effects between treatments. Since all fragments were exposed to identical conditions, except temperature, during the tank portion of the experiment, any differences in the measured variables between bleached and non-bleached control fragments were due to the temperature effects alone, independent of natural seasonal variation. Significant differences between recovery intervals were defined as seasonal differences.

Significant differences in δ^13^C enrichment values among bleached and non-bleached control coral animal host, endosymbiotic algae, and skeletal fractions in the DI-^13^C and ^13^C-rotifer pulse-chase experiments were determined using a 3-factor ANOVA with treatment being fixed with two levels (bleached and non-bleached), tissue being fixed with 3 levels (coral host, endosymbiotic algae, and skeleton), and chase being random with 2 levels (4 and 12 hrs). All data were tested for normal distribution from plots of the residuals versus the predicted values for each variable. Rotifer enrichment data were non-normal and were natural log transformed to achieve normality prior to ANOVA analysis. All statistical analyses were done using SAS software, Version 9.2 of the SAS System for Windows. [Copyright © 1999–2001 SAS Institute Inc. SAS and all other SAS Institute Inc. product or service names are registered trademarks or trademarks of SAS Institute Inc., Cary, NC, USA]. *P*-levels ≤0.05 were considered significant.

With only one DOC concentration value and one δ^13^C-DOC value per treatment per pulse-incubation, statistical analyses were not possible. These data were only qualitatively assessed by visual inspection. Any interpretation of this data carries this caveat.

## Results

### Photosynthesis and Respiration, Chlorophyll a, Energy Reserves, and Calcification

**Figure 2:**
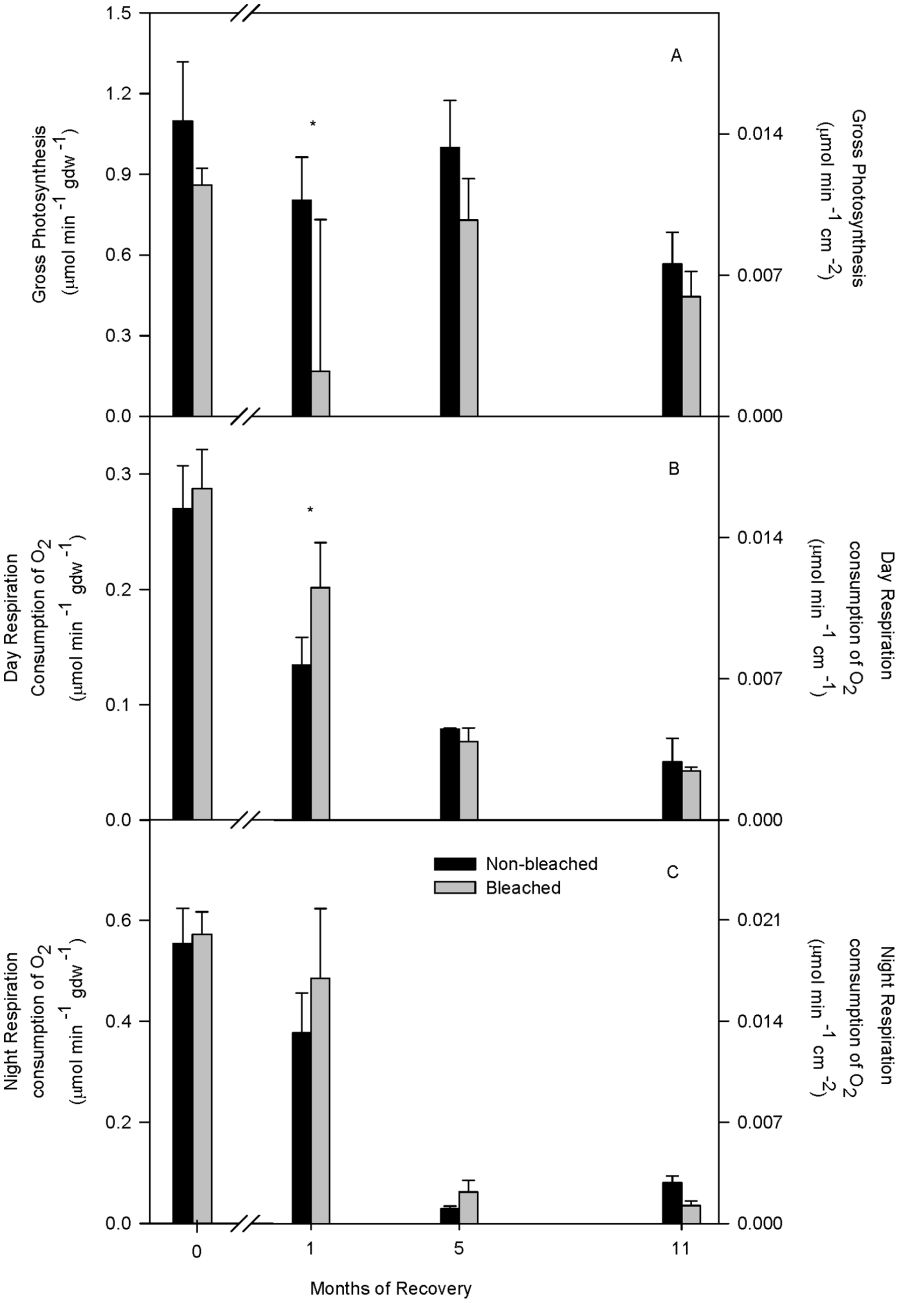
Photosynthesis and respiration rates after bleaching and during recovery. Average (A) photosynthesis rate, (B) day respiration rate, and (C) night respiration rate in non-bleached (black bars) and bleached (gray bars) *Porites lobata* at 0, 1, 5, and 11 months of recovery. Averages for 0 months recovery are standardized to grams of ash-free dry tissue weight (gdw) and are shown ±1 SE from Rodrigues (2006). All other averages are from this study and are reported relative to surface area. Symbols (*) indicate significant differences at p0.05 between means within a recovery interval by a posteriori least-squares mean slice test. Sample sizes for each average were 3.

P rates were 80% lower in bleached fragments compared to non-bleached fragments after 1 month of recovery (Fig. 2A), but did not differ from controls at any other time and had fully recovered to non-bleached control values by 5 months of recovery (Table 1, Fig. 2A). Day and night R did not significantly differ between bleached and non-bleached control fragments overall (Table 1), but post hoc analyses revealed that daytime R was significantly higher in the bleached fragments than in the non-bleached controls after one month of recovery, and had recovered by 5 months of recovery (Fig. 2B,C). Seasonal differences in day and night R were also observed such that both variables were significantly lower after 5 and 11 months of recovery compared to the first month of recovery (Table 1; Fig. 2B,C).

**Table 1:** Results of (A) three students t-tests (0 months recovery) and (B) main and interactive effects of temperature (T), recovery interval (R) (1, 5, and 11 months of recovery), and genotype (G) for average photosynthesis rate, day respiration, and night respiration.

A) Variable				t-statistic	*p-*value	
Gross Photosynthesis				1.04	0.351	
Day Respiration				0.23	0.822	
Night Respiration				0.34	0.741	
**B) Variable**	**Effects**	**df**	**SS**	***F*** **-statistic**	***p-*** **value**	**Tukey**
GrossPhotosynthesis	T	1	0.000096	4.93	0.0571	
	R	2	0.000109	2.78	0.1210	
	T×R	2	0.000032	0.83	0.4722	
	G	4	0.002942	3.73	0.0536	
Day Respiration	T	1	0.000004	1.52	0.2533	
	R	2	0.000139	23.32	0.0005	1>5 = 11
	T×R	2	0.000017	2.97	0.1083	
	G	4	0.000028	2.40	0.1355	
Night Respiration	T	1	0.000006	0.81	0.3940	
	R	2	0.000180	10.78	0.0054	1>5 = 11
	T×R	2	0.000017	1.05	0.3946	
	G	4	0.000124	3.72	0.0538	

Abbreviations: df, degrees of freedom; SS sum of squares of the effect; 1, 5, and 11, recovery interval.

**Figure 3:**
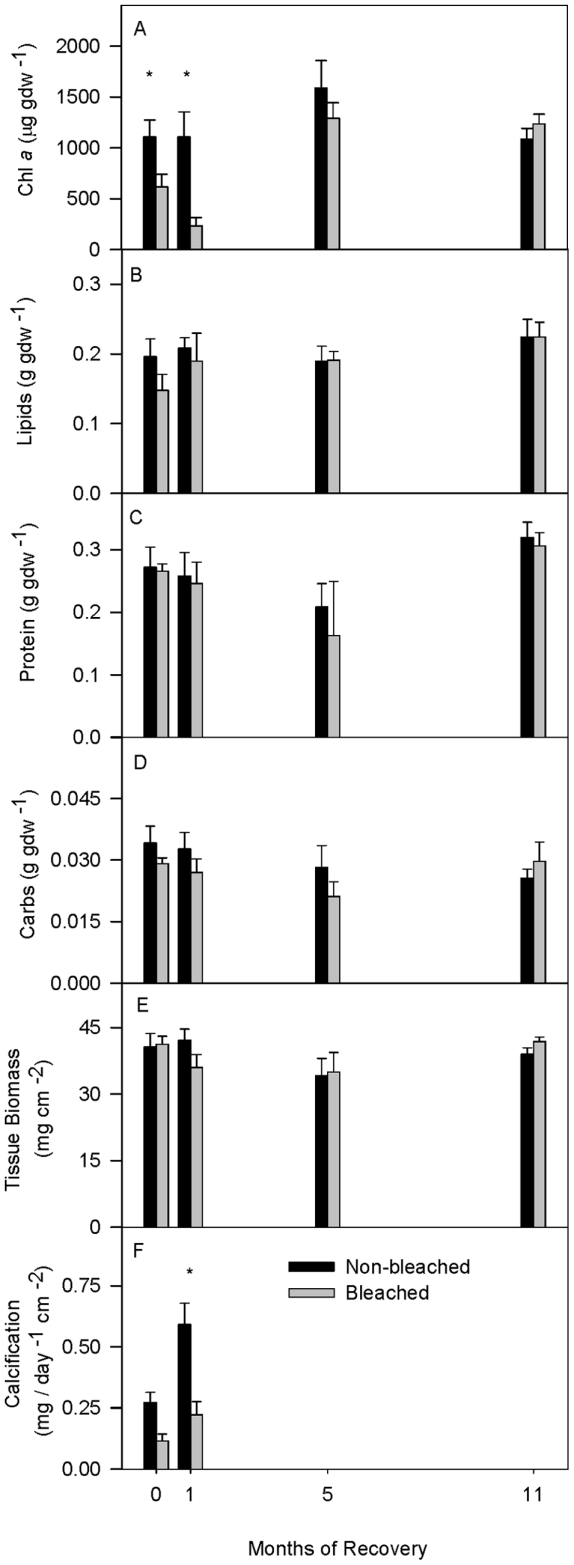
Chl *a*, energy reserves, tissue biomass, and calcification after bleaching and during recovery. Average (A) Chl *a* concentrations, (B) lipid concentrations, (C) protein concentrations, (D) carbohydrate concentrations, (E) tissue biomass, and (F) calcification in non-bleached (black bars) and bleached (gray bars) *Porites lobata* at 0, 1, 5, and 11 months of recovery. Calcification rates were not measured at 5 and 11 month recovery. Averages in A–D are standardized to grams of ash-free dry tissue weight (gdw). All averages are shown ±1 SE. Symbols (*) indicate significant differences at p0.05 between means within a recovery interval by a posteriori least-squares mean slice test. Sample sizes for each average ranged from 3 to 6.

Chl *a* in bleached fragments decreased to 55% and 21% of average non-bleached control fragment values after 0 and 1 month of recovery, respectively, and no longer differed from controls after that (Table 2; Fig. 3A). Seasonal differences in Chl *a* were also observed such that Chl *a* at 0 and 1 month recovery were significantly lower than at 5 and 11 months recovery (Table 2; Fig. 3A). Despite significant decreases in average Chl *a* concentrations and P in bleached corals during the first half of the study, average concentrations of total soluble lipid, soluble animal protein, soluble animal carbohydrate, and tissue biomass did not significantly differ between bleached and non-bleached control fragment averages at any time (Table 2; Fig. 3B–E). Seasonal differences in average soluble animal protein and tissue biomass were also observed such that both variables were significantly lower after 5 months of recovery compared to the other recovery intervals (Table 2). Finally, calcification rates were significantly lower in bleached compared to non-bleached control fragments (Table 2) due in large part to the 36% decline or 0.308 mg CaCO_3_ day^−1^ cm^−2^ decrease in bleached coral calcification observed after 1 month of recovery (Fig. 3F). Calcification rates at 5 and 11 months of recovery were not available.

**Table 2:** Results of main and interactive effects of temperature (T), recovery interval (R), and genotype (G) on Chlorophyll *a*, total lipids, soluble proteins, soluble carbohydrates, tissue biomass, and calcification for all 6 genotypes.

Variable	Effect	df	SS	*F-statistic*	*p-*value	Tukey
Chlorophyll *a*	T	1	331,994	2.17	0.1215	
	R	3	2,077,604	4.54	0.0103	0 = 1<5 = 11
	T×R	3	2,364,563	5.16	0.0058	
	G	5	1,032,249	1.35	0.2720	
Lipids	T	1	0.0030	0.79	0.3820	
	R	3	0.0124	1.06	0.3800	
	T×R	3	0.0033	0.29	0.8336	
	G	5	0.0128	0.66	0.6583	
Proteins	T	1	0.0022	0.35	0.5594	
	R	3	0.0677	3.46	0.0291	5<11
	T×R	3	0.0014	0.08	0.9727	
	G	5	0.0338	1.05	0.4076	
Carbohydrates	T	1	0.0001	1.82	0.1876	
	R	3	0.0002	1.29	0.2961	
	T×R	3	0.0001	0.70	0.5597	
	G	5	0.0006	1.81	0.1415	
Tissue Biomass	T	1	32.8715	1.00	0.3252	
	R	3	296.6213	3.01	0.0460	0<5>11
	T×R	3	79.0360	0.80	0.5025	
	G	5	481.7888	2.93	0.0290	
Calcification	T	1	0.2744	12.87	0.0043	BL<NB
	R	1	0.2029	9.51	0.0104	0<1
	T×R	1	0.0503	2.36	0.1527	
	G	5	0.0168	0.16	0.9729	

Abbreviations: df, degrees of freedom; SS, sum of squares of the effect; NB, Non-bleached; BL, Bleached; 0, 1, 5, and 11, recovery interval.

Of the nine variables presented so far, a genotypic effect was only detected in one (i.e., total biomass) (Table 2) and is therefore not a primary factor influencing these variables.

### Natural abundance stable Isotopes of the Skeleton, Animal Host, and Endosymbiotic Algae

**Figure 4:**
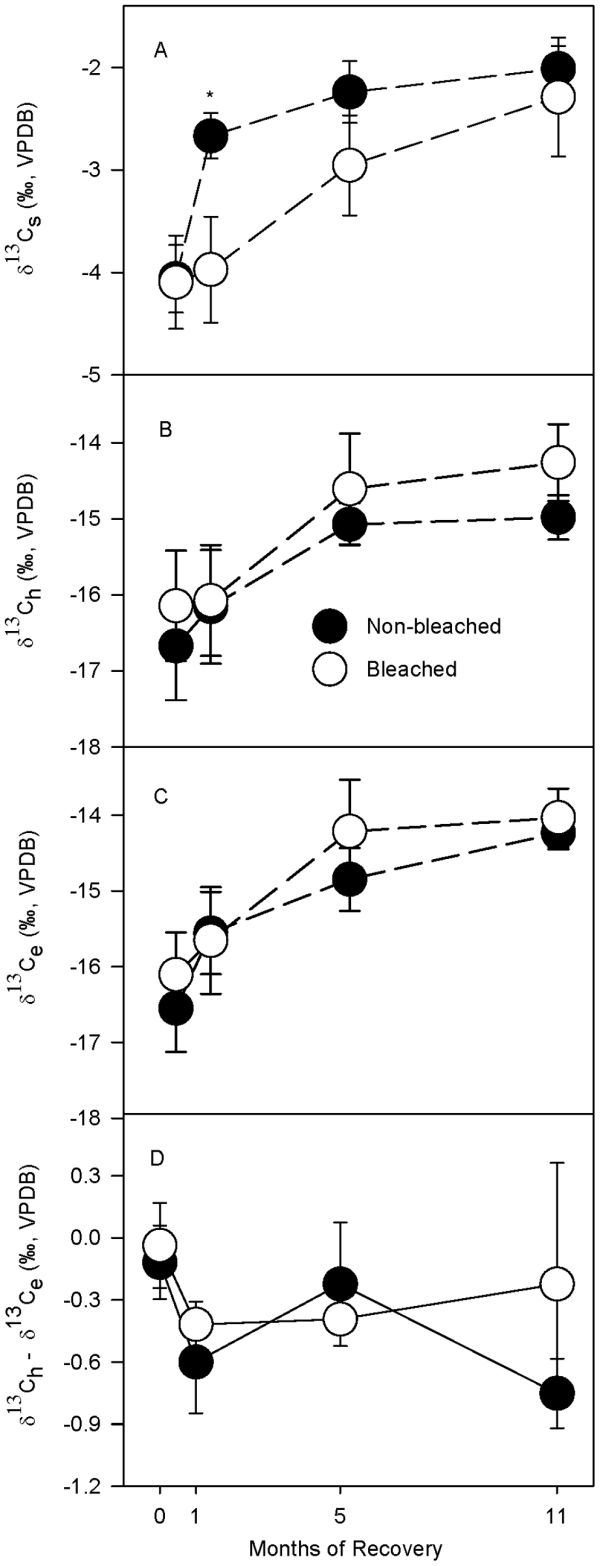
δ^13^C of the skeleton, animal host, and endosymbiont. Average δ^13^C of the (A) skeletal (δ^13^C_s_), (B) host tissue (δ^13^C_h_), (C) endosymbiont (δ^13^C_e_) fractions, and (D) the difference between δ^13^C_h_ and δ^13^C_e_ in *Porites lobata* at 0, 1, 5, and 11 months of recovery. In D, heterotrophy contributes more to the fixed carbon pool when the difference is <0, while photosynthesis contributes more when the difference is >0. All averages are ±1 SE. Symbols (*) indicate significant difference at p0.05 between non-bleached (•) and bleached (○) within a recovery interval using a posteriori least-squares mean slice tests. Sample sizes for each average ranged from 3 to 6.

Average stable carbon isotope values of the skeleton (δ^13^C_s_) were significantly lower in bleached than in non-bleached control fragments (Table 3) with a dramatic decrease of 1.31‰ in bleached coral fragments after 1 month of recovery compared to the non-bleached controls (Fig. 4A). Average δ^13^C_s_ had fully recovered to non-bleached control values by 5 months of recovery (Fig. 4A). Average stable carbon isotope values of the host, endosymbiont, and host minus endosymbiont (δ^13^C_h_, δ^13^C_e_, and δ^13^C_h_–δ^13^C_e_, respectively) did not significantly differ between bleached and non-bleached control fragments at any recovery interval (Table 3; Fig. 4B–D). Seasonal effects were detected such that all three categories of δ^13^C values (i.e., δ^13^C_s_, δ^13^C_h_ and δ^13^C_e_) increased over the course of the experiment (Table 3; Fig. 4). In addition, there was a significant genotypic effect for δ^13^C_s_, δ^13^C_h_ and δ^13^C_e_ (Table 3).

**Table 3:** Results of main and interactive effects of temperature (T), recovery inverval (R), and genotype (G) on δ^13^C_s_, δ^13^C_h_, δ^13^C_e_, and δ^13^C_h_–δ^13^C_e_ for all 6 genotypes.

Variable	Effect	df	SS	*F*-statistic	*p*-value	Tukey
δ^13^C_s_	T	1	3.406	6.64	0.0153	BL<NB
	R	3	6.425	12.53	<0.0001	0<5 = 11
	T×R	3	0.881	1.72	0.1853	
	G	5	11.775	4.59	0.0033	
δ^13^C_h_	T	1	0.129	0.21	0.6519	
	R	3	13.713	7.36	0.0008	0 = 1<5 = 11
	T×R	3	0.673	0.36	0.7811	
	G	5	55.075	17.74	<0.0001	
δ^13^C_e_	T	1	0.000	0.00	0.9523	
	R	3	2.313	12.21	<0.0001	0 = 1<5 = 11
	T×R	3	0.174	0.92	0.4437	
	G	5	3.138	9.94	<0.0001	
δ^13^C_h_–δ^13^C_e_	T	1	0.083	0.32	0.5759	
	R	3	1.488	1.91	0.1501	
	T×R	3	0.514	0.66	0.5835	
	G	5	2.515	1.94	0.1186	

Abbreviations: df, degrees of freedom; SS, sum of squares of the effect; NB, Non-bleached; BL, Bleached; 0, 1, 5, and 11, recovery interval.

**Figure 5:**
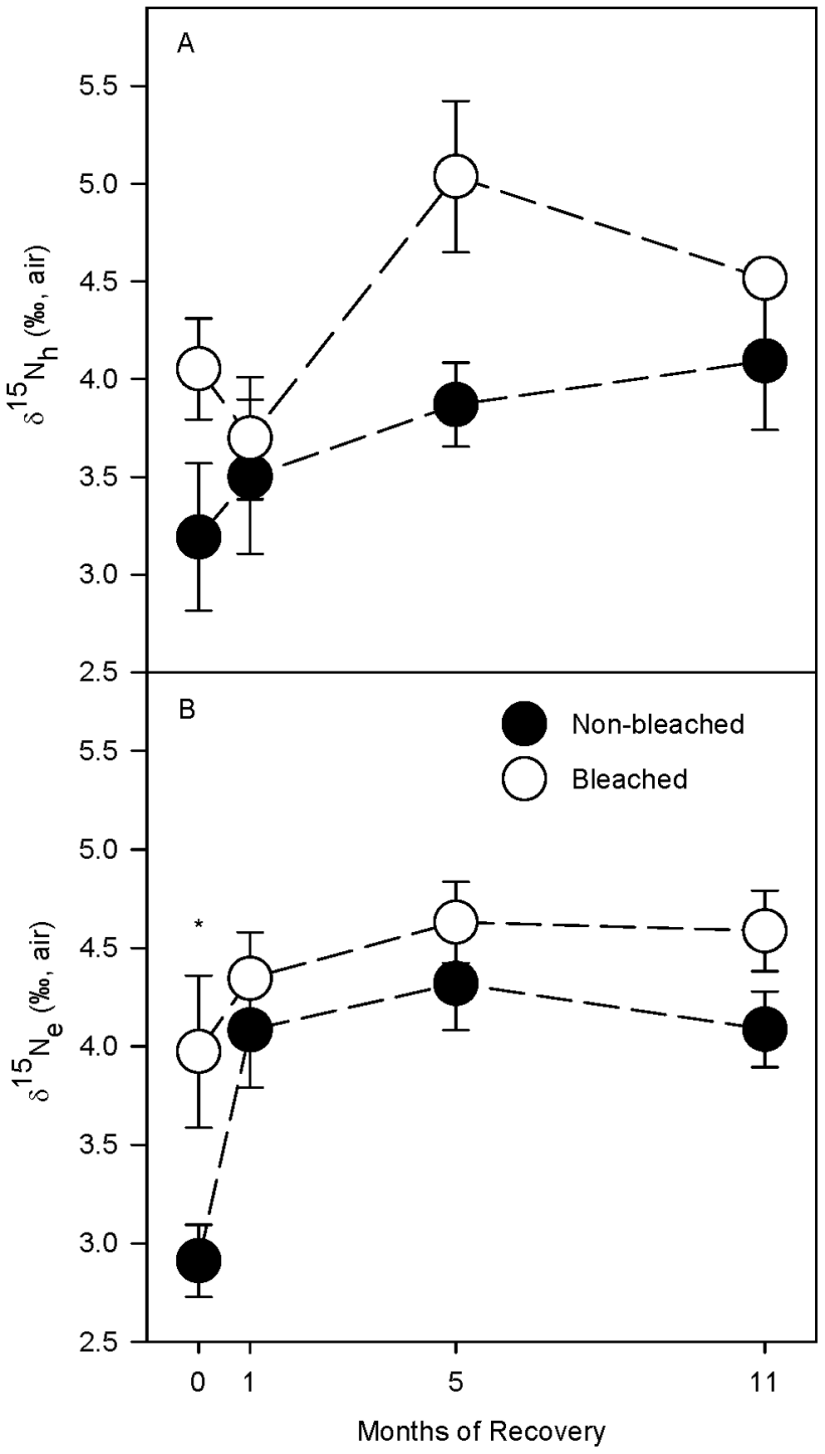
δ^15^N of the animal host and endosymbiont. Average stable nitrogen isotopic (δ^15^N) values for (A) the host tissue (δ^15^N_h_) and (B) endosymbiont (δ^15^N_s_) fractions for *Porites lobata* at 0, 1, 5, and 11 months of recovery. All averages are ±1 SE. Symbols (*) indicate significant difference at p0.05 between non- bleached (•) and bleached (○) fragments within a recovery interval using a posteriori least-squares mean slice tests. Sample sizes for each average ranged from 3 to 6.

Average stable nitrogen isotope values of the host (δ^15^N_h_) were significantly greater in bleached compared to non-bleached control fragments (Table 4), in large part because bleached coral fragments were heavier than non-bleached controls after 0 and 5 months of recovery (Fig. 5A). Average stable nitrogen isotope values of the endosymbiont (δ^15^N_e_) were also significantly heavier in bleached compared to non-bleached controls (Table 4), which was most pronounced after 0 months of recovery when the average bleached δ^15^N_e_ values was 1.76‰ greater than in the non-bleached controls (Fig. 5B). δ^15^N_e_ values in the bleached fragments had fully recovered by 1 month of recovery (Fig. 5B). There was a genotypic effect detected in δ^15^N_e_ but this effect was not detected in δ^15^N_h_.

**Table 4:** Results of main and interactive effects of temperature (T), recovery inverval (R), and genotype (G) on δ^15^N_h_ and δ^15^N_e_ for all 6 genotypes.

Variable	Effect	df	SS	*F*-statistic	*p*-value	Tukey
δ^15^N_h_	T	1	3.644	5.52	0.0258	BL>NB
	R	3	5.049	2.55	0.0752	
	T×R	3	1.317	0.67	0.5803	
	G	5	0.743	0.23	0.9487	
δ^15^N_e_	T	1	2.460	8.00	0.0084	BL>NB
	R	3	7.051	7.64	0.0007	0<1 = 5 = 11
	T×R	3	1.209	1.31	0.2898	
	G	5	4.122	2.68	0.0413	

Abbreviations: df, degrees of freedom; SS, sum of squares of the effect; NB, Non-bleached; BL, Bleached; 0, 1, 5, and 11, recovery interval.

### ^13^C-enrichment experiments

**Figure 6:**
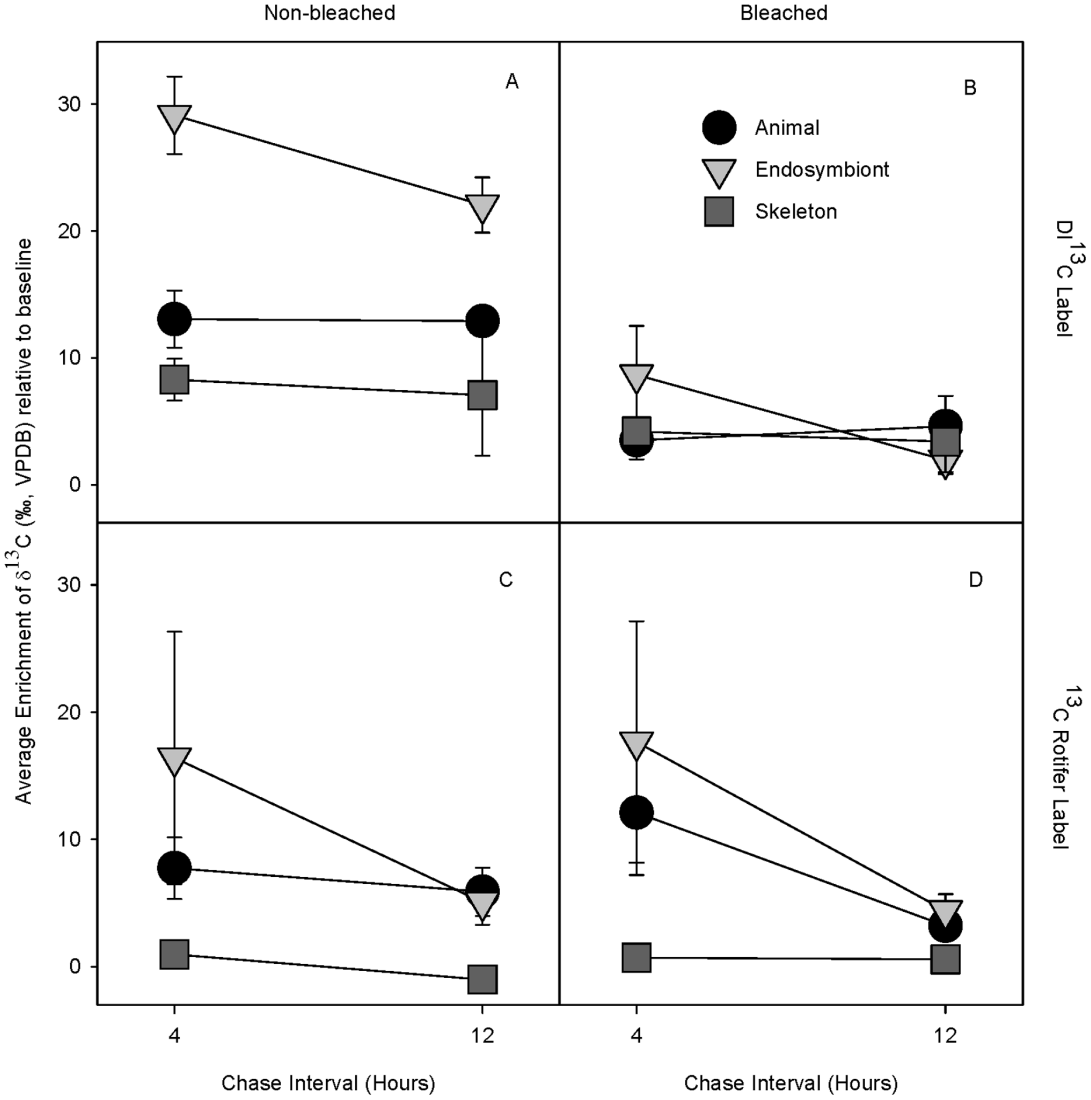
Effects of bleaching on DIC and rotifer uptake and allocation in *P.* *lobata*. Average δ^13^C enrichment (±1 SE) of the animal, endosymbiotic algae, and skeleton of (A, C) non-bleached, (B, D) bleached *Porites lobata* corals during a 12-hr chase following incubation with (A, B) DI^13^C-labeled seawater or (C, D) ^13^C-labeled rotifers. Values are given as enrichment relative to natural abundance baseline values. Sample sizes for each average ranged from 5 to 6 at the 4-hr chase interval and ranged between 2 to 3 at the 12-hr chase interval.

Overall, DIC-^13^C incubated bleached *P. lobata* coral fragments were significantly less enriched in ^13^C than their non-bleached controls due to dramatically lower δ^13^C enrichment levels in the bleached endosymbiotic algae and animal host fractions (Table 5; Fig. 6A,B). δ^13^C enrichment did not significantly differ between any fraction of the bleached and non-bleached coral fragments incubated with ^13^C-labeled rotifers, but did decline significantly over the chase period (Table 6; Fig. 6C,D). In addition, the skeletal fraction was less enriched than either the endosymbiotic algae or the animal host (Table 6; Fig. 6C,D).

**Table 5:** Results of main and interactive effects of temperature (T), tissue fraction (Ti), and chase interval (Ch) on δ^13^C enrichment during a 12-hr chase following an 8-hr incubation with DI-^13^C-labeled seawater.

Effect	Df	SS	*F*-statistic	*p*-value	Tukey
Temperature	1	1261.28	40.23	<0.0001	BL<NB
Tissue	2	707.05	11.27	0.0001	Skeleton = Animal<Endo.
Chase	1	63.63	2.03	0.1615	
T×Ch	1	1.18	0.04	0.8471	
Ti×Ch	2	106.34	53.17	0.1955	
T×Ti×Ch	2	0.34	0.17	0.9946	
T×Ti	2	502.18	8.01	0.0011	Animal = BL<NB
					Endo = BL<NB
					Skeleton = BL = NB

Abbreviations: df, degrees of freedom; SS sum of squares of the effect; NB, Non-bleached; BL, Bleached; Endo. = endosymbiont.

**Table 6:** Results of main and interactive effects of temperature (T), tissue fraction (Ti), and chase interval (Ch) on δ^13^C enrichment during a 12-hr chase following an 8-hr incubation with ^13^C-labeled rotifers.

Effect	df		SS		*F*-statistic		*p*-value		Tukey
Treatment	1	0.013		0.04		0.8523			
Tissue	2	11.767		15.13		<0.0001		Skeleton<Endo. = Animal	
Chase	1	2.226		5.73		0.0221		4>12	
Tr×Ch	1	0.005		0.01		0.9073			
Ti×Ch	2	0.480		0.62		0.5451			
Tr×Ti×Ch	2	0.616		0.79		0.4603			
Tr×Ti	2	0.361		0.47		0.6318			

Abbreviations: df, degrees of freedom; SS sum of squares of the effect; Endo. = endosymbiont; 4–12, chase interval.

**Figure 7:**
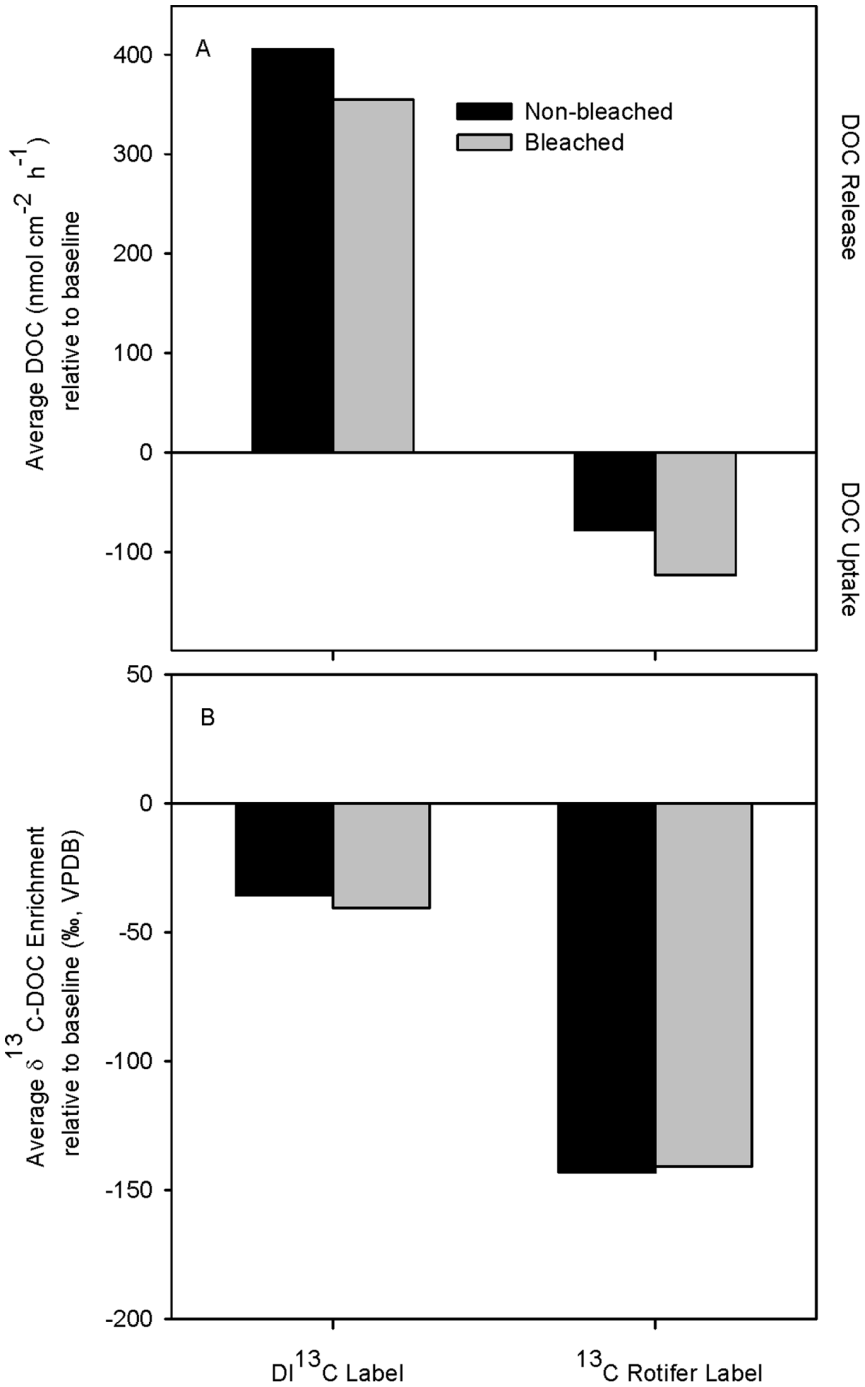
DOC as a potential source of fixed carbon. (A) DOC concentrations and (B) δ^13^C-DOC depletion from ^13^C-labeled DIC or rotifers in bleached (gray bars) and non-bleached (black bars) *Porites lobata* corals relative to their respective baseline controls. Each value in A) was standardized using the total surface area of all 6 incubated coral fragments.

DOC concentration in the DI^13^C control incubation aquaria was 53 µmol l^−1^. In the presence of non-bleached and bleached corals, DOC concentrations more than doubled to 121 and 128 µmol l^−1^, which amounted to coral release rates of 405.5 and 355.4 nmol cm^−2^ h^−1^, respectively (Fig. 7A). DOC concentrations in the ^13^C-rotifer control incubation aquaria was 234 µmol l^−1^. In the presence of non-bleached and bleached corals, DOC concentrations decreased by 10% and 22%, which amounted to a coral DOC uptake rates of −78.1 and −123.1 nmol cm^−2^ h^−1^, respectively. Thus, when exposed to ^13^C-rotifer, bleached corals had DOC uptake rates that were 36% higher than those of non-bleached corals. When incubated in DIC-^13^C labeled seawater, both bleached and non-bleached *P. lobata* coral fragments released DOC to the surrounding seawater that was isotopically depleted relative to the coral-free seawater control (Fig. 7A,B). When incubated with ^13^C-labeled rotifers, both bleached and non-bleached fragments removed DOC from the surrounding seawater leaving behind seawater DOC that was isotopically less enriched than that of the coral-free seawater control (Fig. 7A,B).

## Discussion

### Photosynthesis and Respiration, Chlorophyll a, Energy Reserves, and Calcification

Observed decreases in P rates in bleached *P. lobata* of 80% at 1 month of recovery were consistent with decreases of 67% and 90%, respectively, for the bleached Hawaiian corals, *P. compressa* and *M. capitata* [32] and 57% for *Stylophora pistillata* [58]. However, observed *P. lobata* day R rates were slightly lower than rates of 0.005 to 0.011 µmol O_2_ cm^−2^ min^−1^ reported for *Stylophora pistillata* and similar to rates of 0.003 to 0.006 µmol O_2_ cm^−2^ min^−1^ reported for *Turbinaria reniformis* [59]. Elevated R rates have also been observed in temperature-stressed juvenile *Stephanocoenia intersepta* corals [60], and in some [58], [61], but not all [32], [58] adult bleached corals. Borell et al. (2008) [58] found that fed temperature stressed *Stylophora pistillata* were able to maintain gross P and had higher R rates than starved corals. Therefore, the elevated day R rates observed at 1 month of recovery suggest either there is an increase in heterotrophic C intake and/or repair mechanisms were elevated (Fig. 2B). The overall lack of any decreases in R rates in the initial stages of recovery indicates that bleached *P. lobata* do not reduce metabolic demand to compensate for reduced photosynthetic capacity nor to conserve energy reserves.

Reduced Chl *a* immediately after bleaching and in the initial recovery phase is consistent with that observed during natural [30], [33], [34] and experimentally induced bleaching [32], [62]. Despite significant Chl *a* losses, bleached *P. lobata* were still able to meet 96% of daily metabolic demand photosynthetically [25] and to maintain P rates at 0 months recovery (Fig. 2A). In comparison, bleached *Montipora capitata* and *Porites compressa* only meet 41% and 74% of metabolic demand photosynthetically, respectively [25]. By 5 months, *P. lobata* Chl *a* had fully recovered, coinciding with the recovery of P and R rates (Fig. 2 and 3A). In addition, *P. lobata* in Kaneohe Bay harbor the thermally tolerant sub-clade C15 endosymbiont [63]. Together, these data suggests that, in *P. lobata*, Chl *a* is present in excess and/or that the thermally tolerant endosymbiont enable(s) *P. lobata* to maintain photosynthesis during bleaching (i.e., 0 months recovery) and promotes rapid recovery from bleaching.

Coupled with its strong photosynthetic performance, *P. lobata* maintained all of its energy reserves and tissue biomass when bleached (Fig. 3B–E). This finding is unique to *P. lobata.* In all other studies to date, tissue biomass and/or one or more of the energy reserve variables declines in bleached corals compared to non-bleached corals [24], [30], [32]–[35], [64]. However at the same time as energy reserves and tissue biomass were maintained in bleached *P. lobata*, calcification rates decreased at 1 month recovery (Fig. 3F). Only when Chl *a* concentrations and gross P were both severely impacted did bleached *P. lobata* have reduced calcification rates. The negative effect of thermal bleaching on coral calcification rates has been well documented [36], [65]–[69]. Since calcification in corals is an energy consuming process [70]–[73], bleached *P. lobata* may be compromising their calcification rates to conserve energy reserves and tissue biomass.

### Natural Abundance Stable Isotopes of the skeleton, animal host, and endosymbiotic algae

Depleted δ^13^C_s_ values in bleached *P. lobata* after a month of recovery, followed by full recovery by 5 months, coincided with observed decreases and recovery of both P rates and Chl *a* concentrations at the same recovery intervals (Fig. 2A, 3A, and 4A). Thus in *P. lobata*, δ^13^C_s_ appears to be tightly coupled to P rates of the organism. This is consistent with observations in healthy corals [38], [39]. However, *P. lobata* appears to be the only coral with a predictable bleaching and recovery response in its δ^13^C_s_ signature. Among Hawaiian corals, neither *Porites compressa* nor *Montipora capitata* had reliable δ^13^C_s_ signatures following bleaching [35], [36]. In other regions, *Porites lutea* and *Montastraea annularis* also did not produce reliable δ^13^C_s_ records of bleaching [30], [66], [69], [74]. In contrast to δ^13^C_s_, the δ^13^C_h_ and δ^13^C_e_ values did not differ between bleached and non-bleached *P. lobata* at any time during recovery (Fig. 4B, C); similar to observations in another *Porites* coral, *P. compressa* [36]. This is consistent with observations that *P. lobata* does not increase feeding rates (i.e., a source of isotopically depleted carbon) in response to bleaching [24], [25].

While δ^13^C_h_ and δ^13^C_e_ values appear to be largely influenced by the host feeding regime, coral δ^15^N_h_ and δ^15^N_e_ values appear to be influenced primarily by the endosymbiotic algal physiology. Increased δ^15^N_h_ values in bleached *P. lobata* are most likely due to decreases in endosymbiont concentrations (Fig. 5A) as hypothesized by Rodrigues and Grottoli (2006) [36]. Comparable δ^15^N_h_ values have been observed in non-symbiotic corals [75], [76], and although endosymbiont concentrations were not measured for *P. lobata*, similar δ^15^N_h_ enrichments were documented in bleached and recovering *P. compressa* [36] that had low endosymbiont concentrations. In addition, dissolved inorganic nitrogen (DIN) is isotopically enriched [77] on reefs, is a significant source of nitrogen for endosymbionts [78], and coral DIN uptake promotes endosymbiont population growth and increased Chl *a* concentrations [79]. During recovery, corals require a large influx of DIN to support endosymbiont and Chl *a* recovery. Increases in the rate of DIN incorporation would result in decreased isotopic fractionation, increased incorporation of ^15^N from the nitrogen source, and more enriched δ^15^N_e_ values. Therefore, increased δ^15^N_e_ values at 0 months recovery (Fig. 5B) are potentially indicative of increased DIN incorporation. Increases in δ^15^N_e_ were also reported by Rodrigues and Grottoli (2006) [36] for bleached *M. capitata* and *P. compressa* but only after 1 month of recovery. This suggests that bleached *P. lobata* are able to facilitate endosymbiont recovery faster than both *M. capitata* and *P. compressa* by potentially increasing DIN uptake immediately after bleaching.

### ^13^C-enrichment experiments

Previous studies have established that healthy Hawaiian corals *M. capitata* and *P. compressa* utilize photoautotrophically acquired C for calcification and to meet short-term metabolic demands and calcification, while zooplankton acquired C is used for tissue building [29]. When bleached, however, photosynthetically acquired C assimilation into the skeleton is dramatically reduced, while heterotrophic C assimilation remains unchanged [29]. However, a slightly different pattern was observed in the present study with *P. lobata*. While photosynthetic C continues to be the main source of C for calcification in non-bleached *P. lobata*, the maintenance of the δ^13^C enrichment values over the 12-hr chase period in the animal host and endosymbiont fractions suggest that photosynthetic C is the main source of fixed C for tissue building (Fig. 6A). When bleached, the dramatic drop in the assimilation of photosynthetically fixed C indicates that neither calcification nor tissue building is being supported by photosynthesis (Fig. 6B). As with *P. compressa* and *M. capitata*, heterotrophic C played a significant role in the internal C pools of *P. lobata*. First, the decrease in δ^13^C enrichment values in all three fractions during the ^13^C-rotifer chase intervals indicates that *P. lobata* catabolizes heterotrophically acquired C to meet short term metabolic demand (Fig. 6C,D). Second, the animal and endosymbiont δ^13^C enrichment values were equally enriched indicating the host actively translocates zooplankton derived C to the endosymbiont. Translocation of heterotrophically derived fixed C to the endosymbiont could enhance endosymbiont recovery following bleaching (Fig. 6C,D). Additional study with a larger sample size and a longer chase interval is needed to confirm the interpretation of these data.

The labeling experiments further revealed that the presence of zooplankton altered DOC fluxes in both bleached and non-bleached *P. lobata* corals. In the absence of rotifer feeding, *P. lobata* released isotopically-depleted DOC (Fig. 7, DI-^13^C label). Recent photosynthetically fixed C should be isotopically enriched with respect to stored C reserves due to the DI^13^C labeling. Therefore, both bleached and non-bleached *P. lobata* released DOC produced from existing C-stores and not from newly photosynthesized C (Fig. 7). This is consistent with findings by Tanaka et al. (2008) [80] where the DOC released by two species of corals was derived from previously-synthesized coral organic C. However when *P. lobata* were allowed to feed, DOC was taken up irrespective of bleaching status (Fig. 7). This is contrary to most DOC flux studies to date which have all shown most corals release DOC when healthy [80]–[87] and when bleached [81], [88]. However, it should be noted that none of the corals in the previous studies were fed at any point during the experiments. Given that corals are surrounded by zooplankton naturally on reefs, the net DOC uptake responses detected in this study under the ^13^C-rotifer label scenario are probably more reflective of natural conditions than results reported under feeding-free conditions. Furthermore, it should be noted that the DI^13^C incubations took place during the day while the ^13^C- rotifer incubations were conducted at night. Coral DOC production has been shown to be greater during the day than at night [89], [90], while seawater-borne heterotrophic bacteria continually consume DOC [91]. Therefore, the DOC decrease in ^13^C- rotifer incubations could be interpreted as an overall net consumption by bacteria rather than coral holobiont uptake. However, since the coral DOC uptake/release rates calculated were corrected relative to the control, the contribution of the seawater-borne bacteria on DOC consumption/release is taken into account. Thus, the only other potential source of DOC consumption would be from the coral holobiont itself including its associated bacterial community within the coral mucus layer. To date there are no studies that have distinguished between DOC uptake by the coral animal and uptake by the bacteria within the mucus. Previous studies have hypothesized two possible mechanisms for DOC uptake: 1) DOC uptake in non-bleached corals is the result of heterotrophic microbial activity and not due to active coral host uptake [83], and 2) DOC uptake in bleached corals is a direct result of active coral host DOC ingestion caused by bleaching stress [28], [82]. Irrespective of the uptake mechanism, our DOC concentration and isotopic values taken together and in the context of all of the other measurements in this study show that in the presence of rotifers, which is representative of conditions on reefs where zooplankton are present, both healthy and bleached *P. lobata* take up DOC as a source of fixed C (Fig. 7A,B). Thus, the distinct ability of *P. lobata* to take up DOC as a source of fixed C when in the presence of zooplankton, irrespective of bleaching status, may be one of the key variables that impart resilience in this species to bleaching stress.

### Synthesis: What Makes P. lobata Resilient?

Overall, the results show that *P. lobata* corals appear to have intrinsic traits that may help to buffer them against the negative effects of bleaching which include: 1- compensating physiological mechanisms, 2- inherent biological traits, and 3- fast recovery rates. First, the compensating mechanisms observed here reveal that, during the first month of recovery following bleaching, the animal host compensated for reduced P rates and Chl *a* (Fig. 2A, 3A) by increasing uptake of heterotrophically derived DOC (Fig. 7), and decreasing energy allocated to calcification (Fig. 3F, 6A–B) enabling it to maintain its energy reserves and biomass in the early stages of recovery (Fig. 3). In addition, the endosymbiotic algae were able to sustain normal P rates initially (i.e., 0 months of recovery) (Fig. 2A) and meet nearly 100% of their metabolic demand [24] all while increasing their uptake of DIN (as indicated by the δ^15^N_e_ enrichment in Fig. 5B), facilitating mitotic cell division and endosymbiotic algal recovery [79].

Second, *P. lobata* also has some inherent biological traits that impart resilience to bleaching. *Porites lobata* typically has double or more the amount of tissue biomass of other coral species [24], [34], [64], [92] (this study Fig. 3E) and has some of the highest lipid concentrations found in corals [32], [33], [93] (this study Fig. 3B) – both of which increase its probability of survival from bleaching [15], [27], [64]. In addition, *P. lobata* has a high basal feeding rate [25], can meet 40–50% of its metabolic demand regardless of bleaching status [25], and readily catabolize zooplankton acquired C to meet metabolic demand (this study Fig. 6D) enabling it to limit the consumption of stored energy reserves and maintain tissue biomass when bleached. Finally, *P. lobata* on this reef harbors endosymbionts from the sub-clade C15 [63], which have been shown to be thermally tolerant [94], [95].

Third, recovery in the mounding *P. lobata* appears to be much faster than in other morphological forms of corals. All of the physiological and isotopic variables that were negatively affected by bleaching initially fully recovered within 5 months. In comparison, only three other species of corals have been monitored during long-term recovery (i.e., greater than 6 months) post-bleaching – bleached *M. capitata* took 8 months to recover, bleached *P. compressa* took more than 8 months to recover, and bleached *M. annularis* had not recovered after a year following bleaching [32], [33], [36]. Some key differences in the physiology of these corals can help to explain the differences in recovery rates. First, unlike *P. lobata*, *M. capitata*, *P. compressa*, and *M. annularis* corals catabolized their energy reserves and tissue biomass when bleached and have low basal feeding rates when healthy [29], [32], [33], [36], [96]. However when bleached, both *M. capitata* and *P. lobata* display significant heterotrophic plasticity (i.e., dramatically increases heterotrophic feeding to compensate for decreased photosynthetically fixed C when bleached) by increasing zooplankton feeding rates and DOC uptake rates, respectively [24], [25] (this study Fig. 7A). It is unknown if *M. annularis* can increase its heterotrophic feeding rates when bleached. This suggests that heterotrophic plasticity drives the speed of recovery following bleaching and that species such as *P. lobata* and *M. capitata* have a distinct advantage over other coral species in surviving bleaching events.

Thus, both the host and endosymbiont have complimentary physiological mechanisms and other intrinsic traits that taken together impart resilience to the mounding coral *P. lobata*. Based on our findings, the underlying mechanisms that allows *P. lobata* to maintain its energy reserves and tissue biomass is a result of three key factors: 1- utilization of DOC as a fixed C source, 2- the immediate catabolism of heterotrophically derived C, and 3- harboring the thermally tolerant sub-clade C15 endosymbiont. These findings provide some detailed clues as to why *P. lobata*, and potentially other mounding coral species, seem to survive bleaching events better than branching species. In addition, these findings are one of the first to show bleached corals actively take up DOC and further strengthen the argument that heterotrophic C is critical to coral recovery following bleaching.
